# Efficacy of stem cell secretome loaded in hyaluronate sponge for topical treatment of psoriasis

**DOI:** 10.1002/btm2.10443

**Published:** 2023-02-21

**Authors:** Daniela Carrillo, Natalie Edwards, David Arancibia‐Altamirano, Fabiola Otárola, Cynthia Villarroel, Catalina P Prieto, María Gabriela Villamizar‐Sarmiento, Daniela Sauma, Fernando Valenzuela, José Lattus, Felipe Oyarzun‐Ampuero, Verónica Palma

**Affiliations:** ^1^ Laboratory of Stem Cells and Developmental Biology, Faculty of Sciences Universidad de Chile Santiago Chile; ^2^ Facultad de Medicina y Ciencia Universidad San Sebastian Concepción Chile; ^3^ Advanced Center of Chronic Diseases (ACCDiS), Universidad de Chile Santiago Chile; ^4^ Department of Sciences and Pharmaceutical Technology, Faculty of Chemical and Pharmaceutical Sciences Universidad de Chile Santiago Chile; ^5^ Department of Biology, Faculty of Sciences Universidad de Chile Santiago Chile; ^6^ Dermatology Department, Faculty of Medicine Universidad de Chile Santiago Chile; ^7^ Campus Oriente, Department of Obstetrics and Gynecology, Faculty of Medicine University of Chile Santiago de Chile Chile

**Keywords:** freeze dried bioactive sponge, hyaluronic acid, psoriasis, secretoma, tortuous blood vessels, Wharton's gelatin mesenchymal stem cells conditioned media, γδ lymphocytes

## Abstract

*Psoriasis vulgaris* is an inflammatory disease characterized by distinctive skin lesions and dysregulated angiogenesis. Recent research uses stem cell secretion products (CM); a set of bioactive factors with therapeutic properties that regulate several cellular processes, including tissue repair and angiogenesis. The aim of this work was to evaluate the effect of CM of Wharton's gelatin MSC (hWJCM) in a treatment based on the bioactivation of a hyaluronic acid matrix (HA hWJCM) in a psoriasiform‐like dermatitis (PD) mouse model. A preclinical study was conducted on PD mice. The effect of hWJCM, Clobetasol (Clob) gold standard, HA Ctrl, and HA hWJCM was tested topically evaluating severity of PD, mice weight as well as skin, liver, and spleen appearance. Treatment with either hWJCM, HA Ctrl or HA hWJCM, resulted in significant improvement of the PD phenotype. Moreover, treatment with HA hWJCM reduced the Psoriasis Area Severity Index (PASI), aberrant angiogenesis, and discomfort associated with the disease, leading to total recovery of body weight. We suggest that the topical application of HA hWJCM can be an effective noninvasive therapeutic solution for psoriasis, in addition to other skin diseases, laying the groundwork for future studies in human patients.

## 1 INTRODUCTION

Psoriasis is a common chronic inflammatory skin disease that affects 2%–3% of the adult population. The etiopathogenesis of the disease is multifactorial and some of the key factors involve skin barrier damage, environmental factors, immunological disorders, and genetic predisposition.^1^ There is no known cure for this autoimmune disease, which is associated with several comorbidities such as metabolic syndrome, increased insulin resistance, increased cardiovascular risk, and psychological/physical burdens, all leading to shortened life spans.^2^

There are many clinical variants of psoriasis, the most common form *psoriasis vulgaris* occurs in 80%–90% of all patients.^3^, ^4^ *Psoriasis vulgaris* is characterized by well‐demarcated, scaly, and erythematous infiltrated plaques. While the pathogenesis of psoriasis is not fully elucidated, it is widely recognized that pro‐inflammatory cytokines have a key role in both the development and maintenance of psoriatic lesions. The TNF‐α/IL‐23 and IL‐17 cytokine axes play a central role in this disease. IL‐17 is the hallmark cytokine secreted by T helper 17 cells (Th 17). Recent studies have highlighted a novel role of IL‐17 producing γδ T cells in psoriasis. Innate dermal γδ T cells are the major IL‐17‐producing cells in the murine skin after IL‐23 stimulation and in the imiquimod (IMQ) induced psoriasiform‐like dermatitis mice model (PD). Furthermore, IL‐17 secreting γδ T cells are present with high frequency in human psoriatic skin lesions.^5^ These cells can induce dendritic cell maturation through the production of TNF‐α as well as stimulation of other immune cells due to their antigen‐presenting capacity.^6^

Compared to healthy skin, the cutaneous microcirculation of psoriatic plaques present several histological alterations, characterized by the appearance of tortuous capillaries of glomerular aspect.^7^, ^8^ These alterations have been reported to appear in the first stages of psoriatic plaque development as well as in PD.^9^, ^10^ Importantly, relapse of psoriatic lesions has been suggested to be more likely if vascular alterations persist.^7^ To date, studies focused on the clarification of the angiogenic dependence of psoriasis are scarce.

Conventional treatments for psoriasis include the use of topical agents such as corticosteroids, vitamin D analogs, and retinoid derivatives, among others.^11^, ^12^ Nevertheless, the use of topical agents to treat mild psoriasis skin lesions has an extremely low rate of adhesion,^13^ due to forgetting treatment application, and having an uncomfortable feeling after application due to bad smell and unpleasant consistency. These factors hamper patients' adherence to long‐term treatments, particularly in *psoriasis vulgaris* and mild *psoriasis*.

In a search for treatment alternatives based on cell therapy, stem cells have become a prominent subject in regenerative medicine.^14^ Mesenchymal stem cells (MSCs) are plastic adherent cells that have the ability to differentiate into mesodermal tissue lineages (such as osteoblasts, chondrocytes, and adipocytes) and are characterized by the expression of a specific set of surface markers.^15^ Particularly, in umbilical cords, MSC can be obtained from Wharton's Jelly, a connective tissue rich in collagen, hyaluronic acid and sulfated proteoglycans,^16^, ^17^ and referred to as hWJ‐MSC. These cells have several advantages over adult MSC, including a minimally invasive isolation process, no ethical issues surrounding harvesting, and less tumorigenic capacity. hWJ‐MSC are immature compared to MSC derived from bone marrow, which allows them to proliferate better and faster,^18^ and produce lower immunogenicity.^19^ Immunomodulatory, anti‐inflammatory, and angiogenic properties make hWJ‐MSC particularly relevant for the treatment of autoimmune diseases.

Paracrine signaling of MSC has been identified as the main regenerative mechanism by which these cells exert a beneficial effect on tissue repair. MSC‐conditioned media (also known as secretome) contains a broad range of biologically active molecules. Components of hWJ‐MSC‐conditioned media (hWJCM) are important for regularization of the vascularization process in vivo, in both spatial and temporal terms, for tissue repair and wound healing.^20^, ^21^ hWJ‐MSC express a specific set of trophic factors and cytokines in response to the dynamic requirements of a specific tissue in a homeostatic way. Therefore, hWJ‐MSC and their secretome (hWJCM) might be able to restore the balance of the microenvironment in which they are found, either promoting or inhibiting angiogenic processes. This makes hWJ‐MSC a powerful regulator of the niche and hence has therapeutic potential. Administration of hWJCM has been proven to be as effective as the administration of MSC while avoiding the difficulties associated with cell‐based treatments,^22^, ^23^ potentially providing a superior alternative to biological agents for psoriasis therapy. Nevertheless, a major disadvantage of hWJCM is the instability and short half‐life of the protein content.

Hyaluronic acid (HA) is a component of extracellular matrixes, whose properties depend on the degree of polymerization. HA of high‐molecular weight has anti‐inflammatory properties, while HA of low‐molecular weight has been associated with pro‐inflammatory properties.^24^ In addition to the ability to modulate acute and chronic inflammation in vivo, high‐molecular weight HA possesses an analgesic effect.^25^ HA has been used in patches with micro needles^26^ and sponges, both with possible biomedical applications for the release of drugs. Recently, the creation of HA sponges through a lyophilization process has allowed better handling of the final product assuring high stability, easy storage, and transport.^27^

The low efficacy, side effects, and low compliance for topical treatments in psoriasis patients make the bioactivation of a biomaterial such as HA with hWJCM (containing factors that promote tissue repair and functional angiogenesis) attractive. The goal of this study was to design a cell‐free treatment for psoriasis and testing in a murine model of PD.

## 2 RESULTS

Psoriasis is a multifactorial immune‐mediated inflammatory disease. A pleiotropic effect of soluble factors may be required for proper MSC‐driven immune and angiogenic repair. Confirming previous data from others and our laboratory, proteome array data analysis revealed that there are anti‐inflammatory and both pro‐angiogenic and anti‐angiogenic factors present in the hWJCM that could potentially provide relief for acute PD (Figure S1 and Tables S2 and S3).

An liquid chromatography with tandem mass spectrometry (LS‐MS/MS) was used to provide additional pro‐beneficial anti‐inflammatory molecules among the hWJCM that may account for the therapeutic efficacy of the secretomes. The shared pattern of immunomodulatory‐related proteins among four different hWJCM samples was analyzed by Gene Ontology. Interestingly, 49.8% of the overlapping hWJCM proteins identified from the total number of proteins were related to immunology pathways. The total list of Gene Ontology related to immune modulation is shown in Table S2. Next, each piece of data, listed at least once in Gene Ontology related to the immunology pathway, was displayed in alphabetical order. Additionally, each immunomodulatory factor secreted by hWJMSC was expressed as a percentage of total emPAI protein, with data ranges between 0.002% and 31.47% (Table S3).^28^ Noteworthy, proteins described with decreased inflammation properties, like TMSB4X, VIM, KRT1, and COL1A1 were high in protein abundance in the hWJCM. Our results were also consistent with previous data of local acute immune response analyses, revealing that hWJMSC secrete typical pro‐inflammatory mediators, such as IL‐6, CXCL8, CCL2, and CXCL1. However, the relative abundance of these cytokines was low. Altogether, our results agree with previous studies demonstrating that hWJCM has immunomodulatory potential.^29^

### 2.1 The percentage of IL‐17A‐producing γδ lymphocytes is decreased after repeated hWJCM injections in a PD IL‐17A C57BL/6 reporter line

**FIGURE 1:**
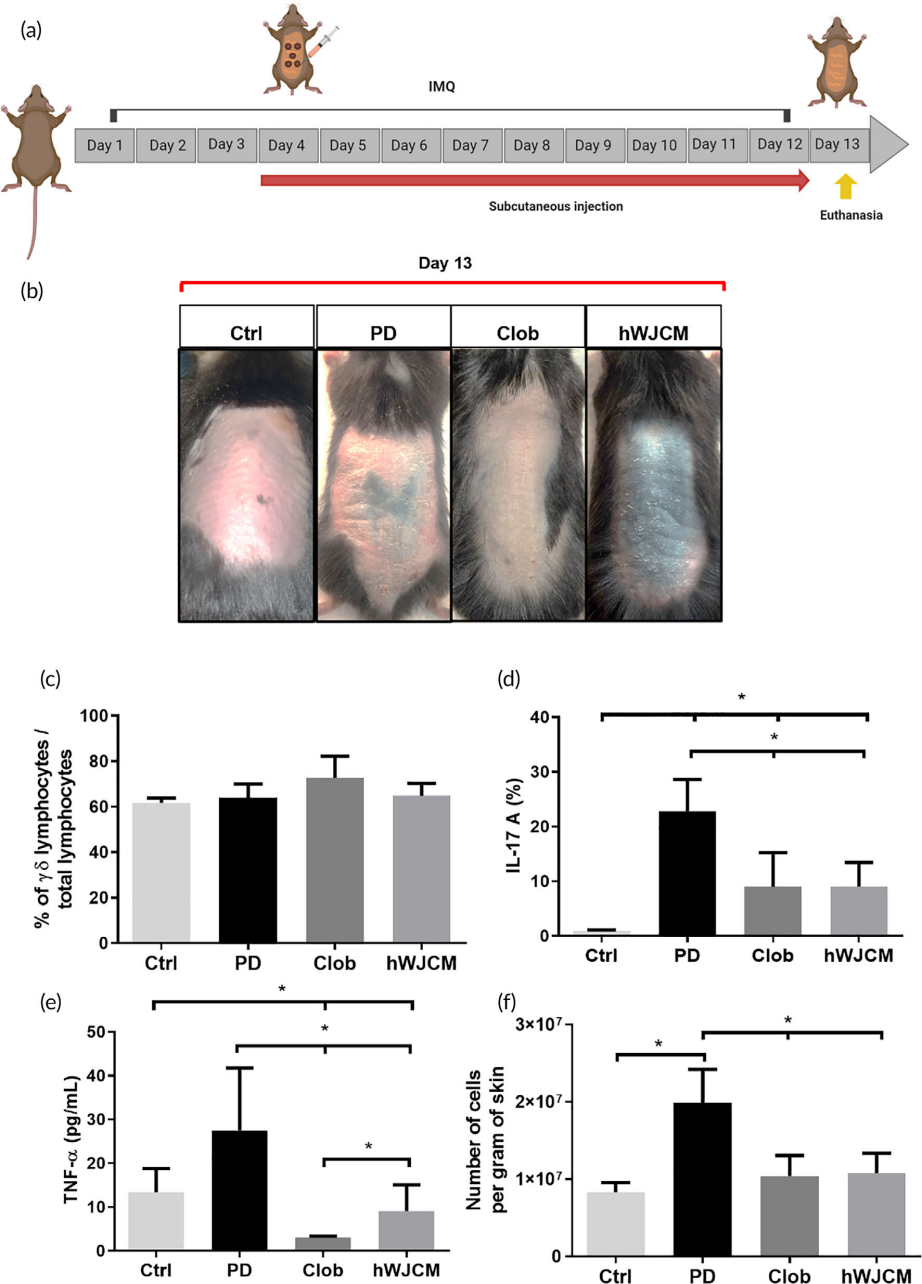
IL‐17A producing γδ lymphocytes in an IL‐17A reporter mice model with PD is decreased after repeated hWJCM injections. (a) Experimental procedure indicating subcutaneous administration of 100 μl of hWJCM for 9 consecutive days, created with BioRender.com. (b) Gross examination. For comparison, gross dorsal skin of mice in each group was photographed at Day 13, 24 h postceasing IMQ treatment. (c) Flow cytometry analysis at Day 13 showing the percentage of γδ lymphocytes in each group. (d) Flow cytometry analysis at Day 13 showing the percentage of IL‐17‐producing γδ lymphocytes in each group. (e) TNF‐α levels present in the skin. (f) Number of cells per gram of skin in mice from each group. Data were analyzed with test for differences per 10,000 paired permutations, *α* = 0.05, *n* = 4.

Here, we used IMQ for induction of PD, known to resemble human plaque‐type psoriasis. The stimulus with IMQ provides the appearance (within 2–3 days after the first application) and maintenance of signs such as erythema, scaling, and thickening and symptoms.^9^ Due to the critical importance of the IL‐23/IL‐17A axis to the pathogenesis of psoriatic disease, we decided to use an IL‐17A C57BL/6 reporter line. Mice were treated for 9 consecutive days with IMQ. Psoriasis Area Severity Index (PASI) scores were determined by evaluating the degree of erythema, thickening, and scaling on the affected dorsal murine skin surface, confirming the phenotypical observations identified in previous reports (data not shown). After verifying the development of severe inflammation in PD, the animal model was used to study the potential therapeutic effect of the novel biologic treatment based on hWJCM. On a first approach, 24 h before PD induction mice were treated with one hWJCM injection and complemented with two additional hWJCM injections at Days 5 and 7 of IMQ treatment (Figure S2A). One day after finishing with the IMQ induction (Day 10), shaved backs were processed to assess the percentage of γδ lymphocytes and IL‐17A producing lymphocytes. Although the percentage of γδ T lymphocytes did not change, hWJCM treatment significantly reduced the percentage of IL‐17‐producing γδ T cells when compared to PD mice (Figure S2B,C). Next, we explored whether a daily injection of hWJCM, starting at Day 4 of a 12‐day IMQ treatment regime, could induce a more pronounced therapeutic effect (see Figure 1a for experimental procedure). Gross examination showed that PD lesions of mice treated with hWJCM are less severe (Figure 1b). While the percentage of γδ lymphocytes in each group did not change (Figure 1c), hWJCM similarly to Clob, considered a gold standard treatment for PD, induced not only a phenotypical reduction in IMQ‐induced skin inflammation but also a significant decrease in cytokines IL‐17A and TNF‐ɑ when compared to PD control (Figure 1d,e). Especially, TNF‐α levels decreased by 89% and 77% as a result of treatment with Clob and hWJCM, respectively. In addition, both Clob and hWJCM treated animals exhibited significantly fewer cells per gram of skin, hence less cellular infiltrate, a hallmark of PD (Figure 1f).

### 2.2 Phenotypical and histological observations confirm skin recovery in PD mice

**FIGURE 2:**
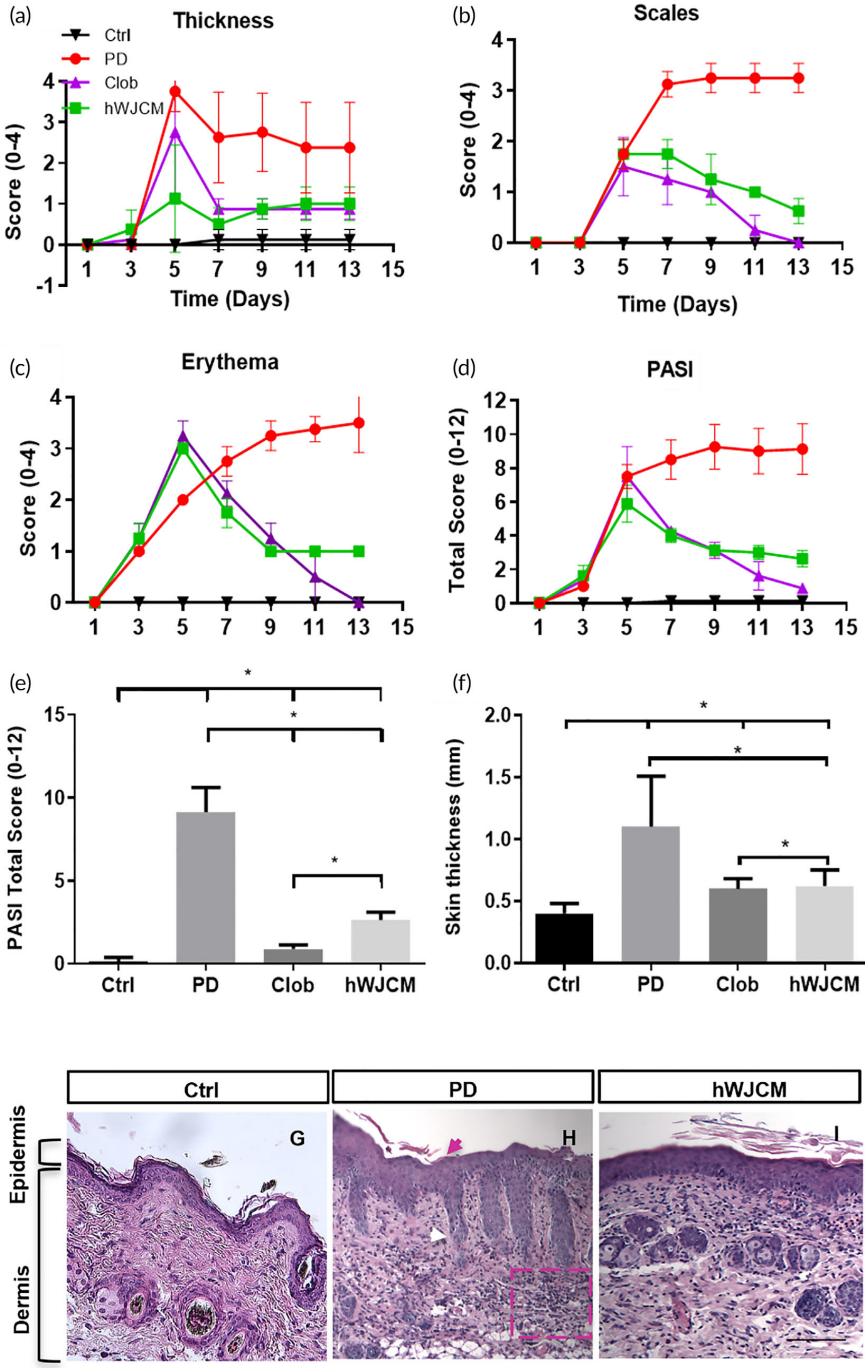
Administration of hWJCM decreases the PASI index in an IL‐17A reporter mice model with PD. (a) PASI scores for skin thickness, (b) PASI scores for scales, (c) PASI scores for erythema, (d) overall PASI scores. Ctrl (black), PD (red) Clob (purple), hWJCM (green). (e) Total PASI score at day 13. (f) Skin thickness (mm). Analysis per 10,000 permutations, *α* = 0.05, *n* = 4. (g) Representative image of histological section of healthy skin (h) Representative image of histological section of skin after 12‐days of PD induction. Typical characteristics of the psoriatic phenotype were noted, such as hyperkeratosis (pink arrow), elongation of epidermal ridges (white arrow) and cellular infiltrate (highlighted in pink box). (h) Representative image of histological section of skin injected with hWJCM once a day for 9 consecutive days in the 12‐day PD induction protocol. Bar = 1 mm (T test, *α* = 0.05, *n* = 15)

The 9‐day hWJCM treatment was able to reduce PD histological characteristics such as thickness of the skin, scaling, and erythema, resulting in a reduced PASI index (Figures 2a–f). Skin thickness in mice treated with Clob was similar to those of hWJCM‐treated mice (Figure 2F). Analysis of H&E‐stained sections from the hWJCM treated dorsal skin was found to be in line with a reduced epidermal thickness in comparison to PD skin (Figure 2g–i).

In summary, our results consistently show that PD was significantly attenuated with local subcutaneous injections of hWJCM. However, injections may cause injury to the site such as pain, bleeding or secondary damage caused by administration of hWJCM. Therefore, we next set out the development of a vehicle scaffold with hWJCM for topical application.

Consistent with previous reports,^30^, ^31^, ^32^ we confirmed that the application of HA lyophilized sponges on diseased skin is able to reduce inflammation. In this work, we demonstrated that BALB/c mice that received the 12‐day PD induction treatment combined with HA sponge administration every day on the back, during the same period, significantly reduced inflammatory cytokine levels, IL‐17A and TNF‐ɑ, returning to values similar to Ctrl mice (Figure S3). Hence, considering a potential synergy between HA sponge and of hWJCM, we explored their combination in the HA hWJCM to be topically administered.

### 2.3 HA hWJCM treatment has angiogenic potential in a chorioallantoic membrane model

**FIGURE 3:**
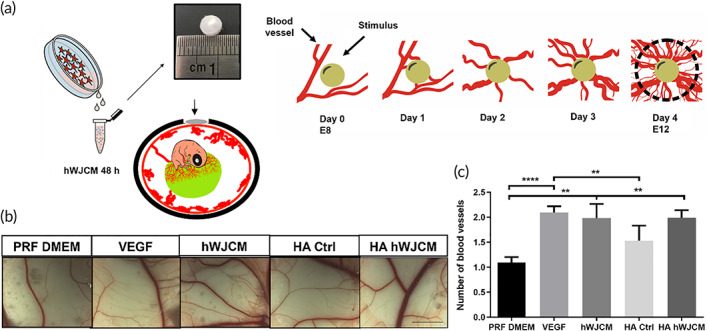
Lyophilized hWJCM in a HA sponge maintains its angiogenic properties. (a) Experimental procedure. (b) Representative images of CAM assay. From left to right: Phenol Red Free (PRF) DMEM negative control, VEGF 50 ng/ml, positive control, hWJCM, HA sponge (control), and HA hWJCM analyzed at E12. Bar = 6 mm. (c) Quantification of the number of blood vessels in CAM assay (one‐way ANOVA, *α* = 0.05, *n* = 10)

The evaluation of the angiogenic potential of a HA hWJCM sponge, obtained after the lyophilization of hWJCM and HA, was determined using a CAM assay. Figure 3a shows the experimental procedure used, along with a representative image of the HA hWJCM sponge. After 4 days of CAM stimulation, an increase in the number of blood vessels compared to the Phenol Red Free (PRF) DMEM (vehicle control) was evident in all experimental conditions (Figure 3b). The quantification reveals significant differences between the PRF DMEM control and the new compositions (Figure 3c). In both hWJCM containing formulations, the total vessel number was similar to those obtained with the positive control VEGF, suggesting a strong angiogenic potential of hWJCM. HA Ctrl had a greater number of blood vessels than PRF DMEM, but there were no significant differences when compared to hWJCM and HA hWJCM.

### 2.4 hWJCM and HA hWJCM improve dorsal skin appearance and decrease the thickness of the epidermis in a PD model

**FIGURE 4:**
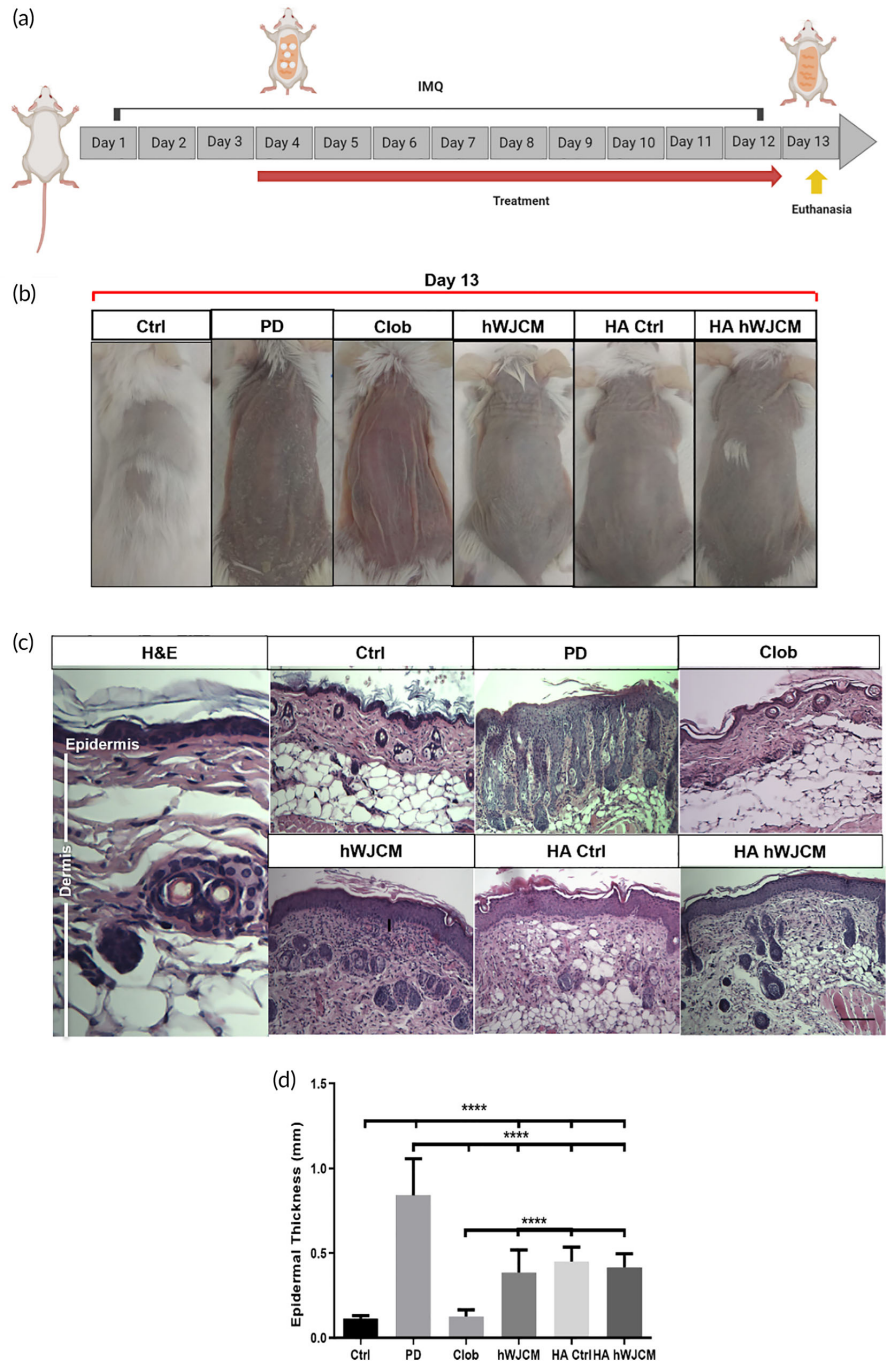
Cutaneous administration of HA hWJCM for 9 consecutive days improves dorsal skin appearance and decreases the thickness of the epidermis in a PD model. (a) Experimental procedure used for the induction of PD. Note that treatments were applied starting on Day 4 in a daily fashion up to Day 12. Clob, HA Ctrl, and HA hWJCM were applied topically while hWJCM was injected subcutaneously. Created with BioRender.com (b) Representative pictures of control mice and mice receiving experimental treatments, Clob, HA Ctrl, HA hWJCM, and hWJCM. (c) Hematoxylin/eosin staining of mouse skin sections at Day 13, Bar = 1 mm. (d) Quantification of epidermal thickness. One‐way ANOVA, *α* = 0.05, *n* = 15

Having established that the HA hWJCM exerted angiogenic effects and maintained the functional properties of the hWJ‐MSC secretome (hWJCM), the next goal was to establish whether the bioactivated polymer (HA hWJCM) would be able to attenuate the signs of IMQ‐induced PD in BALB/c mice. An experimental protocol of 12 days of PD induction was used and four different treatments were performed from the fourth day of induction to evaluate the therapeutic potential of HA hWJCM in comparison to hWJCM injections, HA Ctrl, and Clob (Figure 4a).

Examination of dorsal back skin was performed on Day 13 (24 h after finishing treatments). PD animals showed a high degree of erythema, scaling, and thickening of the skin. Clob, hWJCM, HA Ctrl, and HA hWJCM treatments decreased the appearance of psoriatic lesions, leading to a phenotypic change (Figure 4b). Once the animals were euthanized, skin samples were obtained to analyze in detail the histology of the samples (Figure 4c). In the PD condition, acanthosis, parakeratosis, lengthening of the epidermal ridges and cell infiltrate were clearly observed. Treatment with Clob, hWJCM, HA Ctrl, and HA hWJCM induced a decrease in epidermal hyperplasia and parakeratosis. A considerable decrease of cellular infiltrate in the dermis was found in Clob and HA hWJCM treatments (data not shown). The PD condition presented the highest epidermis thickness followed by HA Ctrl, HA hWJCM, hWJCM, Clob, and Ctrl (Figure 4d).

### 2.5 HA hWJCM treatment decreases both the number of normal and twisted blood vessels and recovers vessel diameter in a PD Model

**FIGURE 5:**
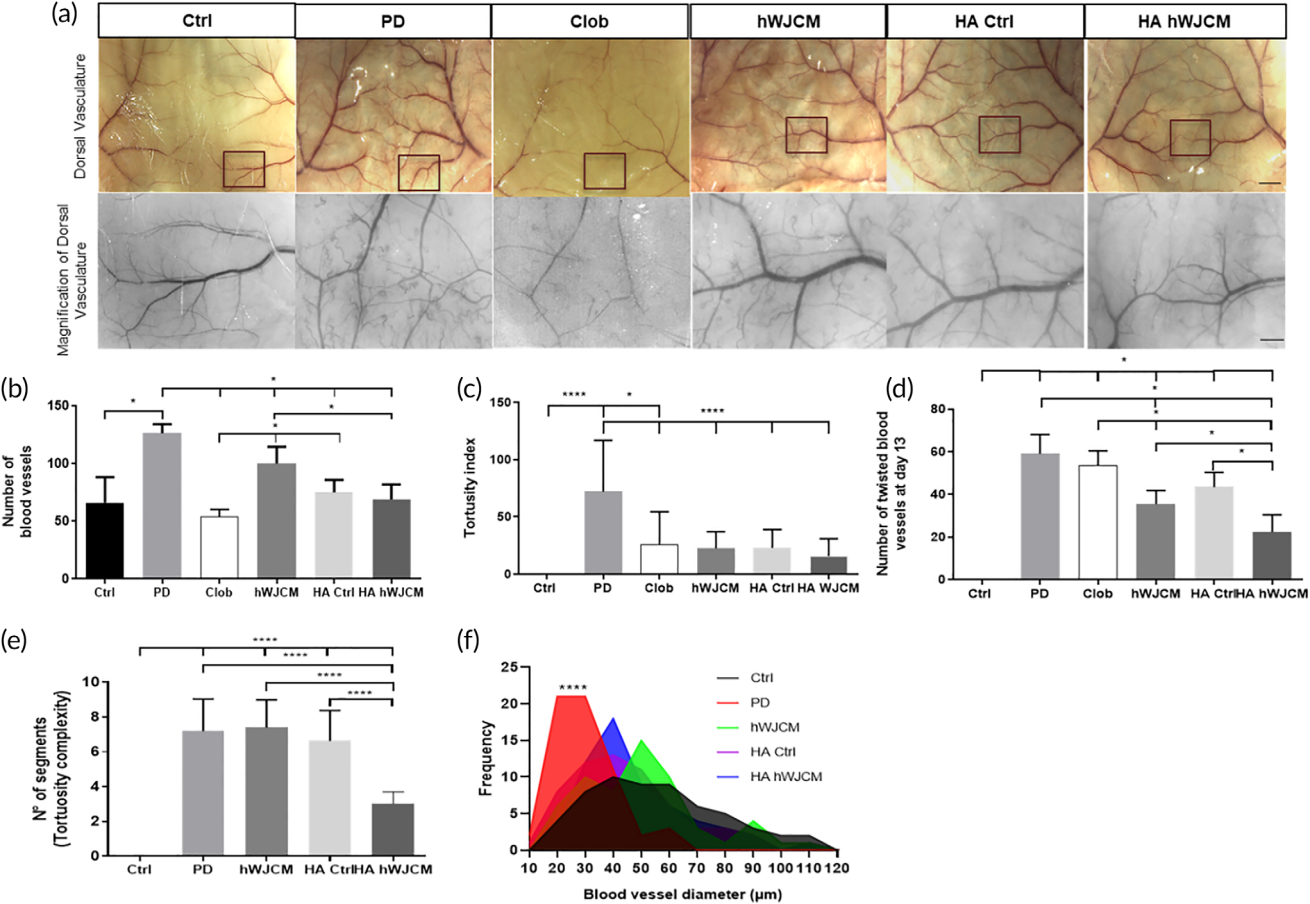
The HA hWJCM treatment decreases both the number of normal and twisted blood vessels and recovers vessel diameter. (a) Low‐ and high‐power images (inset highlighted) of representative skin sections (4 cm × 4 cm) at Day 13 after receiving the indicated treatment. Highlighted inset shows blood vessels derived from main “dorsal branch” (an area of approximately 3 mm × 4 mm). Bar = 2.5 mm for low‐power images, Bar = 0.5 mm for high power. (b) The number of blood vessels in Clob‐, hWJCM‐, HA Ctrl‐, and HA hWJCM‐treated groups. Mann–Whitney test, *α* = 0.05, *n* = 3 (*n* = 4 for Clob). (c) Tortuosity index in Clob‐, HA Ctrl‐, HA hWJCM‐, and hWJCM‐treated groups. One‐way ANOVA with repeated measures; *n* = 60. (d) Number of twisted blood vessels at Day 13 in each treatment group. Mann Whitney *α* = 0.05, *n* = 3. (e) Tortuosity complexity of each treatment group. One‐way ANOVA with repeated measures, *α* = 0.05, *n* = 30 for each group. (f) Distribution graph for vessel diameter and the vessel frequency in each treatment group. Kolmogorov Smirnov test, *α* = 0.05, *n* = 60 for each measurement

Morphological changes described in blood vessels of psoriatic patients suggest dysregulated angiogenesis resulting in the formation of aberrant vasculature. Although tortuous vessels have been observed in psoriasis, they have not been studied in most of the PD mice models. Prior to obtaining skin sections for histological analysis, we acquired images from the dorsal back skin, to observe, analyze and compare in greater detail the microcirculatory alterations of the blood vessels in all the experimental conditions (Figure 5a). Figure S4 illustrates tortuous microvessels and the quantification criteria used for abnormal vessel assessment. Consistent with the literature, after induction of PD, we found an increase in vascularization and formation of tortuous and twisted blood vessels with reduced vessel diameter. The number of blood vessels decreased with all topical treatments (Figure 5b). Surprisingly, topical treatment of PD mice with HA Ctrl and HA hWJCM reduced the tortuosity index drastically, similarly to that found after hWJCM injection or Clob treatment (Figure 5c). The most noticeable difference in the number of twisted blood vessels was identified between PD mice and HA hWJCM‐treated mice. Clob treated and PD mice displayed similar numbers of twisted blood vessels at the end of treatment. After analyzing the tortuosity complexity of tortuous blood vessels as the total number of segments for each vessel, we observed that the vasculature of HA hWJCM treated mice was less complex than PD, hWJCM, and HA Ctrl mice (Figure 5e). The small diameter of new vessels produced as a consequence of PD induction returned to normal levels after treatment (Figure 5f). Of note, it was not possible to establish vessel diameter or tortuosity complexity for the Clob condition due to the extreme thinning of vessels after the steroid application.

Taken together, these findings show that HA hWJCM was the only treatment that improved the structure of PD blood vessels by multiple criteria, recovering overall the vasculature.

### 2.6 Topical administration of HA hWJCM and hWJCM improves PD severity, extent and welfare

**FIGURE 6:**
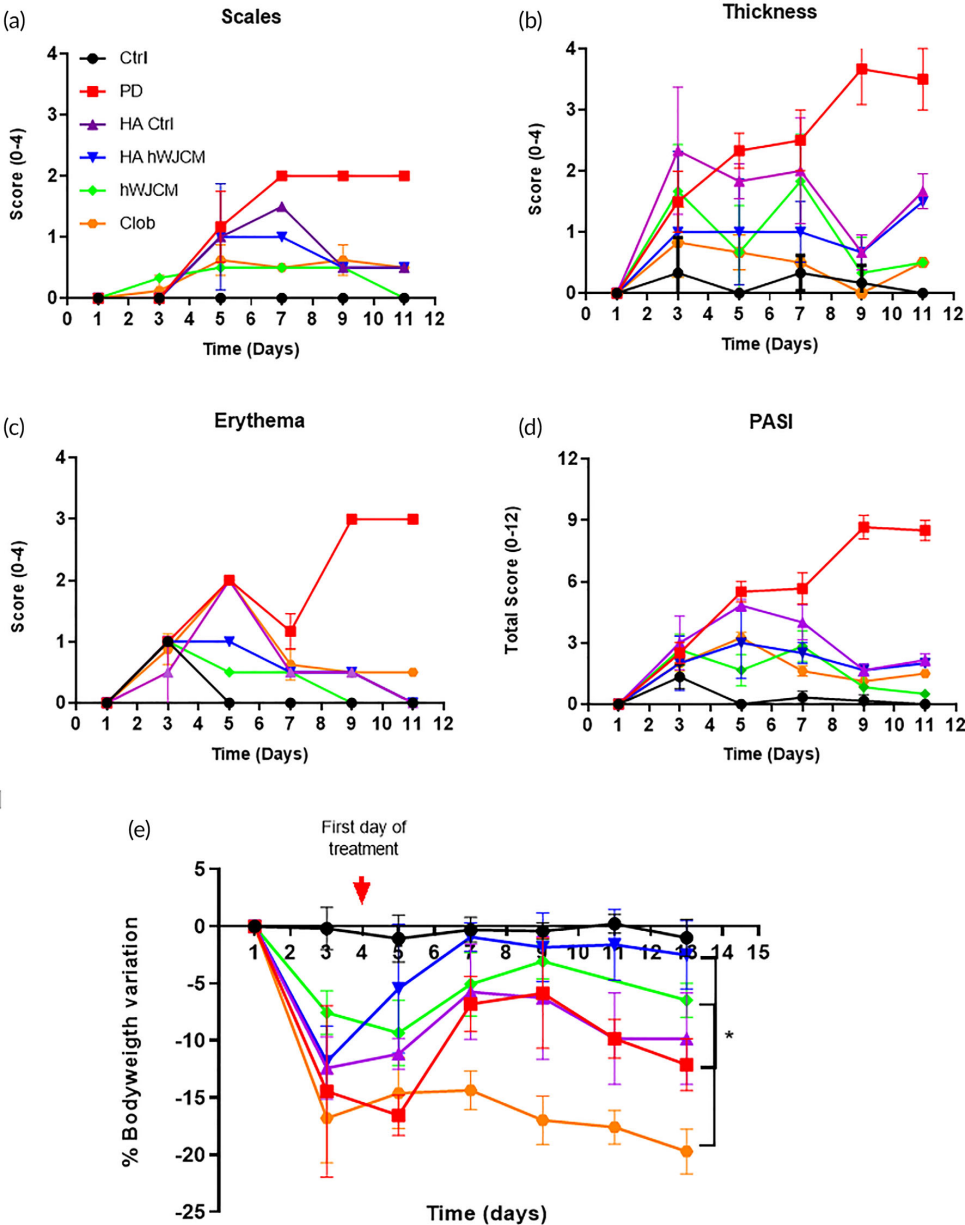
Cutaneous administration of HA hWJCM and hWJCM for 9 consecutive days improves PD animal welfare. Measurement of the PASI index score of the skin on the backs of BALB/c mice treated as indicated. (a–c) Each parameter was evaluated quantitatively; skin thickness, scales, and erythema. (d) Total PASI score Mann–Whitney Test, *α* = 0.05, *n* = 3 (*n* = 4 for Clob). (e) Percent bodyweight variation. Mann–Whitney Test, *n* = 3, *α* = 0.05. Ctrl (black), PD (red), HA Ctrl (purple), HA hWJCM (blue), hWJCM (green), Clob (orange)

PASI score is a widely accepted tool used to measure the severity and extent of psoriasis. Scales, skin thickness, and erythema contribute to the PASI index. Scores from the treatment groups are illustrated in Figure 6a–d. As presented in Figure 6d, on the last day of evaluation (Day 12), the condition with the highest total PASI score was PD (8.5 ± 0.5), followed by animals treated with HA Ctrl (2.17 ± 0.29), HA hWJCM (2 ± 0), Clob (1.5 ± 0), hWJCM (0.5 ± 0) and finally, control shaved Ctrl mice (0 ± 0).

The percent reduction for hWJCM, Clob, HA Ctrl, and HA hWJCM was 94.12%, 82.35%, 74.51%, and 76.47%, respectively. We found that after the first application of IMQ, PD mice lost weight. This result is expected and coincides with reports in the literature, since IMQ induces strong skin inflammation and sores with the consequent discomfort of the animals, which affects their feeding capacity. Importantly, under HA hWJCM treatment mice began to gain weight and, on the last day of treatment, animals practically recovered their original weight (−2.51% vs. −0.99% of the Ctrl group). No other treatment resulted in weight gain. After administration of Clob, a dramatic weight loss was observed, even greater than diseased untreated mice (−20.37%). Mice treated with hWJCM also experienced weight loss (−6.47%), but an interesting trend of recovering is noticeable. Mice treated with HA Ctrl lost weight at a similar level to PD mice (−9.84% and −12.11%, respectively). All data of bodyweight variation are summarized in Figure 6e and individual values are presented in Table S1. We suggest that the better welfare for HA hWJCM treated animals is a consequence of the improved therapeutic effect of the formulation, resulting in more comfort and increased water and food intake.

Psoriasis is defined as a state of systemic inflammation, which involves other organs besides the skin, such as the spleen. To determine whether topical treatments contribute to the overall recovery of PD in BALB/c mice we also measured both splenic and PASI index. As expected, the size and weight of the spleen were markedly enlarged in PD mice compared to Ctrl. The ratio of spleen weight to bodyweight was still significantly increased after all treatments with exception of the Clob‐treated group, which showed a decreased splenic index when compared to the Ctrl group (Figure S5). The latter indicating that both topical treatments (HA Ctrl and HA hWJCM) as well as hWJCM injections act locally, improving the skin condition, but do not enter in circulation (i.e., do not reduce the systemic inflammation, inherent to PD).

## 3 DISCUSSION

### 3.1 hWJCM reduces the percentage of γδ T lymphocytes that produce IL‐17 in a murine model of PD

Psoriasis is among the most common T‐cell‐mediated disorders. Recent research has focused on IL‐17‐producing cell types, such as the Th17 cells, γδ T‐cells, and CD8 T‐cells. Our results indicate that attenuation of psoriatic lesions in PD mice is mainly due to a significant decrease in key pro‐inflammatory cytokines such as TNF‐α and IL‐17A. However, we cannot rule out that the various bioactive compounds found in hWJCM could act on more than one signaling pathway that relieve symptoms or affect its chronicity. hWJCM may decrease the presence of other highly expressed key agents such as IL‐1, IL‐6, IL‐23, IFN‐α and IFN‐γ, IL‐12, and IL‐22 as has been described for PD mice after UC‐MSC administration.^33^

The role of γδ T lymphocytes as the main source of IL‐17A at early stages of psoriasis has been established, both in murine and human models.^5^ To date this is the first study to report the reduction of the percentage of γδ T lymphocytes that produce IL‐17 as an effect of hWJCM administration in a murine model of PD (Figure 1). There are some in vitro studies supporting, in some extent, our results, since a co‐culture of peripheral blood mononuclear cells from patients with spondyloarthritis treated with UC‐MSC inhibits the production of IL‐17.^34^

Since the frequency of γδ T cells does not vary with the different treatments, we propose that the hWJCM is reducing IL‐17 production by this population of cells. IL‐17 production by γδ T cells is mainly driven, even in the absence of TCR engagement, by cytokines such as IL‐1 and IL‐23, which amplify IL‐17 responses. Factors contained within the hWJCM reduce IL‐1 and IL‐23 production by other cells (such as dendritic cells) therefore reducing IL‐17 production by γδ T cells.

### 3.2 HA hWJCM shows angiogenic activity in a CAM assay

It is well known that storage of secretomes represent a complication when projected as a therapeutic solution because it requires very specific conditions in order to preserve the stability.^35^, ^36^ In this article, the preservation of the biological activity in secretomes is promoted by the extraction of their aqueous medium by lyophilization while increasing the interaction of the components with the dry matrix of HA.^37^, ^38^, ^39^ In addition, our strategy allows an easier transport and storage, allowing a wider availability.

Once the HA hWJCM biopolymer was obtained, a CAM assay was carried out to assess whether the angiogenic potential previously described for hWJCM^21^ is maintained in the new composition and after a lyophilization process. The loss of functional properties of therapeutic components could be caused by conformational changes of lyophilized proteins in addition to unwanted transformations of active compounds and/or excipients.^40^, ^41^ Regarding the CAM assay (Figure 3c), it could reasonably be expected to obtain an additive/synergistic effect when hWJCM and HA are combined, but this is not appreciated in the experiment. On the one hand, this could be explained due to the achievement of maximum appreciable effect in this CAM model when applying the selected stimuli (similarly to the effect achieved with the positive control VEGF). On the other hand, the result could be attributed due to a modification in the release kinetic of the actives from the formulation, a slower and prolonged release of the components could modulate the angiogenic response. There are several articles reflecting a modulation of the release of the actives when entrapped in hyaluronan matrices.^42^, ^43^, ^44^

It should be noted that although the HA Ctrl stimulus generated an increase in the number of total vessels, this was not considered significant. The diameter of the blood vessels under VEGF, hWJCM, and HA hWJCM treatment, showed a significant increase compared to Ctrl (data not shown); suggesting an effect on the maturation of the blood vessels or proliferation of endothelial cells.

### 3.3 Recovery of severity, extent and welfare of PD‐treated animals as a consequence of the HA hWJCM treatment

Although the psoriasiform dermatitis induction model with Imiquimod allows recapitulating the main clinical and histological manifestations of skin with psoriasis, it is not a chronic study model, since to maintain the PD condition, IMQ must be applied daily. If application is stopped, the mouse recovers and the clinical and histological manifestations disappear. One of the strategies referenced in the literature is to apply IMQ and treatment on the same day in order to maintain PD for longer.^45^, ^46^ Administration of Clob, hWJCM, HA Ctrl, and HA hWJCM decreased the PASI index in PD mice; hWJCM having the greatest effect (Figure 6). This is possibly due to the more invasive method of administration (subcutaneous injection) and to the aqueous nature of hWJCM, which would contribute to a reduction in the dehydration caused by IMQ and would therefore improve skin appearance. Although topical noninjectable administration of the secretome was not performed in this study, it has been described that the topical application of bone marrow MSC CM of rats decreases the inflammation caused by IMQ after only 3 days of treatment; reducing the thickness of the skin to the same level as fluocinolone, a powerful corticosteroid.^47^ Interestingly, despite the previously reported pro‐angiogenic profile for hWJCM, in a PD context, a decrease in the degree of erythema was observed in individuals treated with either hWJCM or HA hWJCM. The latter is particularly relevant since it would indicate that in a psoriatic context in vivo, characterized by permanent inflammation, the anti‐angiogenic and anti‐inflammatory components of hWJCM take on a greater role. Rather than attributing therapeutic activity to a single factor present in the secretome, it is important to keep in mind that the final outcome of the conditioned media will depend on the local niche of action and the balance between components that promote angiogenesis and those that inhibit it.^44^ The healing effect performed by hWJMSC is most presumably because they act as trophic mediators that help to create a regenerative microenvironment by secreting a variety of therapeutic factors. In accordance, here we showed that the hWJMSC contains skin healing‐related factors that include, among others, immunomodulatory proteins, both angiogenic and anti‐angiogenic factors, which becomes extremely relevant for proper vascularization and its fine regulation, in spatial and temporal terms in vivo. After the application of the formulations, we presume that the effect could be promoted due to a prolonged tissular distribution of the active molecules to a variety of targets (such as blood vessels). In the case of the topical administration of sponges, the disrupted skin barriers could provide a better permeation of the actives to the targets. In every case, a long‐lasting effect could be favored. Considering that hWJCM may vary within the same donor and/or with different donors, to promote reproducibility for each batch, we analyzed the most expressed proteins in the secretomes. The selected molecules are TMSB4X, VIM, and FSTL1. In addition, and as a second control, we also checked in all hWJCM samples a similar set of proteins that could be involved in the immune response (Table S3). Finally, to tightly control the protein content between hWJCM and HA hWJCM, a solution comprising 3.3 ml of hWJCM (obtained from three different WJMSC cultures) and 26.4 mg of HA were combined to obtain 13.2 ml. From this suspension, 100 μl was extracted to obtain each sponge, which corresponds to ~1.2 μg of protein/sponge. As presented in this article, the selected secretome, in addition to containing a variety of soluble factors (Figure S1 and Tables S2 and S3), contains extracellular vesicles (EVs). These vesicles show a hydrodynamic diameter between 100 and 400 nm, a negative zeta potential between −5 and −11 mV, and a concentration between 0.4 and 4.6 ✕ 10^9^ vesicles/ml (see the methodology section for details about the characterization). Thus, the therapeutic effect should be a reflection of both kinds of components and following a different release kinetic from the HA matrix.^42^, ^43^, ^44^ In terms of the EVs from MSC, there exists evidence that they could alleviate psoriasis‐associated inflammation by the reduction of IL‐17.^48^ In addition, topical application of EVs in a mouse model of IMQ‐induced psoriasis resulted in the reduction of inflammation.^49^

Clob treatment was able to reduce the psoriasiform phenotype. Analysis revealed that the skin did not look healthy as in HA Ctrl, HA hWJCM, or Ctrl mice. In fact, Clob‐treated mouse skin looked extremely wrinkled and thin. The results obtained for HA Ctrl and HA hWJCM indicated that the reduction of the PASI index for both treatments was similar. Importantly, mice receiving HA hWJCM treatment had an almost complete weight recovery after just 4 days of treatment, unlike the PD, Clob, hWJCM, and HA Ctrl mice. Animals treated with HA Ctrl did not maintain weight, decreasing at almost the same level as PD mice. Even though high‐molecular weight HA has been reported to decrease pro‐inflammatory effects and have an analgesic effect,^24^, ^25^ the failure here to cause body weight gain suggests that HA Ctrl alone was not sufficient to minimize the pain associated with PD, unlike HA hWJCM treated mice, a matter which deserves further research. The increased well‐being resulting from the HA hWJCM treatment is possibly due to a synergy of the therapeutic effects of its components. Notably, the Clob treatment group experienced major body weight loss, even more pronounced than the PD condition, in agreement with previous data.^50^

In contrast to Ctrl mice, classic psoriatic manifestations were seen in all PD animals and disappeared with concomitant Clob application, the gold standard topical treatment. Importantly, HA Ctrl, hWJCM, and HA hWJCM treatments also controlled these manifestations, possibly due to the treatment acting on keratinocytes, regulating their proliferation, thereby reducing the thickness of the epidermis. HA Ctrl, despite having high‐molecular weight, can penetrate the epidermis and dermis as previously reported.^51^ No parakeratosis was observed with HA hWJCM treatment, and unlike with HA Ctrl and hWJCM treatments, this was accompanied by a decrease in cellular infiltration in the dermis (Figure 4c). These results confirm the presence and synergistic effect of immunomodulatory factors in the hWJ‐MSC secretome when combined with HA that could decrease the recruitment of cells of the immune system attracted to the injured skin due to chemoattractants secreted by dying keratinocytes or aberrant behavior triggered by some stressor.^1^

### 3.4 HA hWJCM regulates the angiogenic process in PD

Aberrant angiogenesis has been described in diseases such as cancer and psoriasis. This would explain the initial increase in erythema in PD, with a thickening of the main blood vessels and the emergence of tortuous capillaries,^52^ in addition to numerous small‐diameter branches that are more permeable and lack cells, such as pericytes and muscle cells, capable of providing support.^53^ It is very important to reduce the aberrant angiogenic process, as tortuous vessels reduce or alter blood flow and are able to generate ischemia in distal areas. The finding that HA hWJCM regulates the angiogenic process is extremely relevant (Figure 5). Patients treated with cyclosporine and etanercept (systemic agents capable of modulating the angiogenic process) for 3 months or 24 weeks, respectively, do not present erythematous squamous lesions after completing treatment.^7^, ^8^ However, the regression from a tortuous to a normal vasculature does not occur in either case.^7^, ^8^, ^54^ These data are in striking contrast to what was achieved with HA hWJCM after only 9 days of treatment. This suggests that HA hWJCM could be accelerating wound healing repair in PD lesions. It is known that the phenomenon of angiogenesis in psoriasis precedes hyperplasia of the skin^53^ suggesting that, if the vasculature of the diseased skin does not return to normal once the treatment has finished, a recurrence of the distinctive lesions is likely to occur.^7^, ^55^ The regulatory effect of HA hWJCM suggests that it could treat other conditions that course with an aberrant angiogenic process including the presence of tortuous vessels such as varicose veins, psoriasis, and hemorrhoids or skin conditions such as rosacea, acne, bullous epidermolysis, atopic dermatitis, among others. HA hWJCM could also be beneficial to treat aberrant neovascularization in diseases such as cancer or diabetic retinopathy.

## 4 MATERIALS AND METHODS

### 4.1 Cell isolation and cultures

Cultures were generated and expanded from fresh umbilical cords (processed no more than 1 day after the date of delivery), following standard procedures implemented in Dr. Palma's laboratory.^21^, ^56^ All primary cultures of WJ‐MSC were used between passages 2–5 as previously characterized.^21^

hWJ‐MSC were seeded in 60 mm diameter plates (p60), to 80%–90% confluence, in Dulbecco's modified Eagle's medium (DMEM, Life Technologies) supplemented with 10% fetal bovine serum (FBS, Biological Industries) and antibiotics (100 U/ml penicillin/streptomycin, Thermo Scientific). Cultures were maintained in a humidified atmosphere containing 5% CO_2_ at 37°C.

### 4.2 hWJCM collection and angio‐proteomic profile studies

Cell density was evaluated under a microscope such that 100,000 cells were seeded per p60 plate. hWJ‐MSC cultures, grown to 80%–90% confluence, were serum‐starved in PRF DMEM for 48 h, and hWJCM was freshly collected, immediately frozen in liquid nitrogen, and stored at −80°C until further use. At least three different hWJCM, obtained from distinct hWJ‐MSC cultures, were pooled together for experimental procedures. hWJCMs were not concentrated prior to their use. hWJCMs assayed through a human Angiogenesis Array kit (Catalog # ARY007, R&D Systems Inc., Minneapolis, USA), used according to the manufacturer's instructions. Spots were detected by chemo‐luminescence and intensity was quantified by densitometry (ImageJ, NIH, USA). The levels of each factor were measured in duplicates and normalized to internal controls provided by the assay.

### 4.3 LS‐MS/MS analysis of hWJCMs

After performing a mycoplasma, test a proteomic analysis was conducted in four different representative hWJCM samples. Briefly, the protein content of each sample was purified and quantified in order to obtain 200 ng of secretome tryptic peptides to be performed by LS‐MS/MS.^57^ Protein interaction networks were identified with STRING database helper to visualize a core analysis of the immunology biological functions.^58^

### 4.4 Characterization of EVs content in hWJCMs

The hydrodynamic diameter and zeta potential of the EVs in hWJCMs were determined by dynamic light scattering and laser Doppler anemometry using a ZetaSizer NanoZS (Malvern Instruments, UK). The samples were analyzed as received and by triplicate. The determination of EVs concentration was performed by nanoparticle tracking analysis in a NanoSight NS300 (Malvern Instruments). The samples were analyzed as received and a minimum of five videos (1 min each one) of the particles moving under Brownian motion were captured by the NanoSight. The videos were then analyzed for size distribution and particle concentration using the built‐in NTA v 3.0 software (Malvern).

### 4.5 HA Ctrl and HA hWJCM bioactive scaffold preparation

For the sponge elaboration, a starting solution of 1.6 mg/ml HA (Mw ~330 kDa, Inquiaroma, Spain) and hWJCM was prepared. The 26.4 mg of HA was dissolved into 13.2 ml of Milli‐Q water at room temperature and then mixed with 3.3 ml of conditioned media for a final volume of 16.5 ml. The resulting mixture (HA hWJCM) was transferred into 96‐well plates (100 μl/well). The samples were frozen at −20°C for 24 h and freeze‐dried in a FreeZone 1 freeze‐dryer (Labconco, USA). Control HA sponges (HA Ctrl), without hWJCM, were produced using the same process.

The protein concentration was measured in freshly collected hWJCM (concentration ranged from 40 to 80 μg/ml). In order to avoid protein content variability between secretomes and sponges, a solution comprising 3.3 ml of hWJCM (obtained from three different WJMSC cultures) and 26.4 mg of HA were combined to obtain 13.2 ml. From this suspension, 100 μl was extracted to obtain each sponge, which corresponds to ~1.2 μg of protein/sponge.

### 4.6 Establishment of the psoriasiform‐like dermatitis in mouse models (PD) and general experimental design

Eight to 12‐week‐old BALB/c mice of both sexes, and C57BL/6‐IL17A males, purchased from Jackson Laboratories (C57BL/6‐Il17atm1Bcgen/J), were obtained from the animal facility of the Universidad de Chile and housed under strict specific pathogen‐free conditions at 25°C ± 2°C and 45% relative humidity, with a 12‐h light/dark cycle. Mice had access to food and water ad libitum. All experiments adhered to the principles of the declaration of Helsinki and were approved by Institutional and Bioethical Use Committees (Universidad de Chile, protocol number 18117‐FCS‐UCH).

Mice were divided into groups according to the experimental design. The control group (Ctrl) was shaved mice, or shaved mice receiving Vaseline (IMQ's vehicle). Mice in all the remaining groups received a daily topical dose of 62.5 mg of commercially available imiquimod (IMQ; 5%, Labimiq ITF‐labomed) on the shaved back for either 9 or 12 consecutive days to establish a model of IMQ‐induced PD.

The studied treatments were either hWJCM repeated injections (for detailed protocol, see Figures 1a and Supl. 2A) or Clobetasol (Clob), HA Ctrl, and HA hWJCM sponges. The sponges (five sponges of 5 mm in diameter per animal) were applied along the back of the mouse. Each sponge was placed with a fine tip tweezer on a drop of distilled water on the mouse skin.

Figures 4, 5, 6 refer to several aspects related with the same animal experiment; from real measurements of the skin in millimeters using a caliper (Figure 4d), to graphics representing PASI score evaluation (Figure 6). Figure 6b represents the skin thickness under an international accepted score (from 0 to 4). All these figures help us to concomitantly understand the animal model and results after applying the stimuli.

### 4.7 Scoring severity of skin inflammation

The histological features of PD in mice are similar to psoriasis: erythema, scaling, and inflammation were measured. To score the severity of skin inflammation, a modified scoring system, developed based on PASI, was adopted.^59^ Every other day, erythema and scaling were scored independently on a 4‐point scale: 0, none; 1, slight; 2, moderate; 3, marked; 4, very marked. The thickness of the inflamed skin was measured using a caliper and a conversion of millimeters to a value (on a scale from 0 to 4) was applied. Skin thickness was also used to assess treatment success and was included in the total PASI score.

### 4.8 Histology

Shaved, excised dorsal mouse skin was fixed in 10% neutral‐buffered formalin, dehydrated and embedded in paraffin by conventional methods. Then 8–10 μm skin sections were cut from formalin‐fixed paraffin‐embedded blocks and transferred to glass slides. Sections were stained with hematoxylin–eosin (H&E) and trichrome staining using standard procedures. All samples were photographed with a BX51 fluorescence microscope (OLYMPUS, USA) equipped with a Moticam 2500 digital camera (10× magnification). Epidermal thickness was measured as the average of 15 image skin section measurements of each study group. Five measurements were made on each image of skin section along the length of representative H&E‐stained skin sections using ImageJ software.

### 4.9 Quantification of IL‐17A and TNF‐α in skin samples

For these experiments, the C57BL/6‐IL17A GFP reporter mice were used. We stained gamma‐delta lymphocytes using the gd TCR antibody (clone GL13 from Biolegend) and analyzed the GFP+ IL‐17‐producing cells within the gate of TCR gd+ cells. The percentage of γδ T lymphocytes and IL‐17A‐producing γδ T lymphocytes among total CD45+ cells was quantified by flow cytometry. To measure cytokine levels in skin tissue, 8 mm skin biopsy punches were obtained from the central dorsal skins of mice, after PD induction and experimental treatments. The biopsies were cultured as explants in 96‐well plates (200 μl of DMEM 1% penicillin/streptomycin, 24 h). The concentration of IL‐17A and TNF‐α cytokines was quantified in the supernatant medium by flow cytometry (CBA Cytometric Bead Array, FACSCanto II [BD Biosciences]).

### 4.10 Evaluation of γδ T lymphocytes and IL‐17A‐producing γδ T lymphocytes

PD was induced on the shaved dorsal skin of C57BL/6‐IL17A reporter mice for either 9 or 12 consecutive days. Initially, the administration of hWJCM was performed on Days 0, 5, and 7; 100 μl of hWJCM were administrated as subcutaneous injections, distributed in five spots on the back, concomitant to PD induction in the 9‐day protocol. For the 12‐day protocol, the fourth day of PD induction was completed in the morning and was combined with a daily dose of 100 μl of hWJCM, distributed in five spots on the back, injected every afternoon. On Days 10 and 13, respectively, skin samples (~2 cm × ~1.5 cm) were obtained from both control and treated animals. The skin was deposited in 1 ml of RPMI medium with 5 mg/ml of collagenase IV and 5 μg/ml of DNAse, subsequently cut into small fragments of 2 mm × 2 mm, and incubated for 90 min at 37°C. Then, the skin was mechanically disaggregated between two coverslips and the cell suspension was filtered through a 40 μm cell strainer. Cells were centrifuged at 400 *g* for 5 min and resuspended in 200 μl of PBS. Cells were labeled with anti‐CD45 and anti‐TCRγδ antibodies (Biolegend, USA). We stained gamma‐delta lymphocytes using the gd TCR antibody (clone GL13 from Biolegend) and analyzed the GFP+ IL‐17‐producing cells within the gate of TCR gd+ cells. The percentage of γδ T lymphocytes and IL‐17A‐producing γδ T lymphocytes among total CD45+ cells was quantified by flow cytometry.

### 4.11 Chicken CAM assay

A CAM assay was performed for the in vivo evaluation of the angiogenic potential of hWJCM, HA Ctrl, and HA hWJCM. Briefly, fertilized chicken eggs (Rock iso, Agricola Chorombo, Chile) were incubated at 38.5°C with constant 75% humidity. At embryonic day 1 (E1), 2 ml of albumin was extracted from each egg and a round window (2 cm^2^) was created on E4. On E8, the CAM vasculature was photographed; subsequently, each experimental condition was placed on top of the CAM. For each condition, 10 eggs were used. The CAM assay included testing 20 μl of the following conditions: PRF DMEM (negative control), VEGF 50 ng/ml (positive control) and hWJCM. In addition, sponges (5 mm of diameter) of either HA Ctrl or HA hWJCM were evaluated. The stimuli were positioned between the intersection of two big blood vessels and a coverslip of 12 mm was placed on top. The results of the stimulation were evaluated at E12 by means of photographs of the CAM area on which the coverslip was deposited, using a 0.8 magnifying glass and an HD IC80 digital camera (Leica, Heidelberg, Germany). In order to improve the visualization of vessels, 3 ml of a 1:1 solution of whipping cream and bidistilled water was injected under the CAM before photographing each egg. For quantification, all blood vessels that intercepted the coverslip edges and the total number of vessels within a 6 mm radius of the sponges at E12 were considered. The results were normalized based on the number of vessels at E8.

### 4.12 Evaluation of the angiogenic effect on PD skin

To identify and quantify the changes generated in the dermal vasculature as result of the different treatments, BALB/c mice were euthanized and skin sections of the shaved back (~2 cm × ~2 cm) were obtained from each group. The total number of vessels and their thickness were evaluated, as well as their morphology. For quantification, images were captured with a Leica MZ125 magnifying glass, associated with the Leica IC80 HD camera, AverMedia RECentral software, followed by analysis using the ImageJ software.

### 4.13 Tortuosity quantification

Multiple photographs were taken of the dorsal vasculature of mice with magnifications of 0.8, 1.6, and 4×, always zooming at the center of the vasculature branches. Tortuous vessels were considered as thin vessels that had a spiral or S shape as observed at 4× magnification.

The tortuosity index of twisted blood vessels was obtained using a specific formula considering the ratio between the length of the blood vessel curvature (C) and the shortest distance between its start and end points (L), given by a straight line.^60^ Complexity of tortuous vessels was considered as the number of segments of a specific twisted vessel. This method was based on a published protocol used to quantify dendritic arborization.^61^ Measurements were performed by two independent counters. Quantification was made using ImageJ software.

Due to image resolution and excessive blood vessel thinness, it was not possible to establish vessel diameter and Tortuosity complexity for the Clob condition.

### 4.14 Evaluation of the therapeutic effect of Clob, hWJCM, HA Ctrl, and HA hWJCM on animal's welfare

Animals weighing approximately 20 g each were used for this study. During induction of PD, the weight of the mice was evaluated together with the PASI index. Records were made at Days 1, 3, 5, 7, 9, and 11 of PD induction, prior to administration of indicated treatments (Table S1).

### 4.15 Estimation of splenic index

The spleen from each mouse was isolated and weighed. Splenomegaly was evaluated by calculating the ratio of spleen to body weight of the studied mice.^62^, ^63^

### 4.16 Data Analysis

All statistical analyses were completed using the Graphpad Prism V5.0 and R 2.12.2. software. Data analyzed are presented as the arithmetic mean of the parameter of interest and standard deviation of the mean and were analyzed for statistical significance using a D'Agostino‐Pearson normality test. In case of a normal distribution, an ANOVA test with a significance level of 0.05 (*p* = 0.05) followed by a Tukey test was performed. In the absence of a normal distribution, a nonparametric Mann–Whitney test, Kolmogorov Smirnov or permutation analysis was used. Experiments were performed in triplicate with an experimental *n* ≥ 3. Differences between groups were considered nonsignificant when *p* ≥ 0.05 (ns), significant when *p* < 0.05 (*), highly significant when *p* < 0.01 (**), and extremely significant when *p* < 0.001 (***) and *p* < 0.0001 (****).

## 5 CONCLUSIONS

hWJCM injections contribute to tissue repair in a model of PD and topically applied HA hWJCM is capable of regulating the clinical manifestations of the psoriasis pathology in a murine model. Regulation is not limited to physical and superficial appearance of the skin; morphological, angiogenic, and immunological changes can be appreciated at a histological level. This was reflected in a decrease of more than 75% in the PASI index after 9 days of treatment with HA HWJCM, which in turn, decreased the suffering of the sick animals and increased their general welfare.

These findings suggest that HA hWJCM could be a comfortable, simple, effective, economic, and safe formulation to treat *psoriasis vulgaris* in patients compared to the current options on the market. HA hWJCM could even serve to alleviate comorbidities such as nonalcoholic fatty liver disease and psoriatic arthritis since it could decrease the surrounding pro‐inflammatory factors that exacerbate these conditions. Another point in favor of HA hWJCM administration could be the local and controlled release of bioactive compounds. As psoriasis is a disease of multifactorial origin, the proposed treatment would have the advantages of presenting various compounds capable of acting in a coordinated manner to regulate the angiogenic, immunological and morphological skin manifestations that characterize psoriasis. In order to validate our proposal formulation with hWJCM, studies with patients are required. Among critical analyses to be obtained, we underline: safety, efficacy, and posology. In addition, through the analyses of the microvasculature in patients, the relationship between its normalization and the efficacy could also be confirmed.

Despite being necessary to treat psoriasis, there is low percentage of adherence for topical treatments and 39%–73% of patients do not use medication as prescribed.^13^ Of note, as a lyophilized product, HA hWJCM can be stored in a simpler way than hWJCM, providing a longer shelf life in a cool, dry place with low chances of contamination, resulting in the better handling by patients. HA hWJCM treatment would also have the advantage of being comfortable for the user since, during administration, the sponge comes into contact with the humid skin and disperses instantly without leaving the skin greasy and smelling bad. Therefore, an increase in treatment adherence would be expected. In summary, our preclinical findings are promising, laying the groundwork for future studies in patients, not only affected from psoriasis, but also suffering from different types of dermatoses and diseases that course with aberrant neovascularization.

## AUTHOR CONTRIBUTIONS

**Daniela Carrillo:** Data curation (lead); formal analysis (equal); investigation (lead); methodology (equal); writing – original draft (equal). **Natalie Edwards:** Data curation (equal); formal analysis (equal); investigation (lead); methodology (equal); writing – review and editing (supporting). **David Arancibia‐Altamirano:** Conceptualization (equal); methodology (equal); validation (equal). **Fabiola Otárola:** Conceptualization (equal). **Cynthia Villarroel:** Data curation (supporting); writing – review and editing (supporting). **Catalina P Prieto:** Data curation (supporting); formal analysis (supporting); writing – review and editing (supporting). **María Gabriela Villamizar‐Sarmiento:** Investigation (equal); methodology (equal); writing – original draft (equal). **Daniela Sauma:** Conceptualization (equal). **Fernando Valenzuela:** Data curation (equal); formal analysis (equal). **José Lattus:** Data curation (equal); formal analysis (equal). **Felipe Oyarzun‐Ampuero:** Conceptualization (lead); formal analysis (lead); funding acquisition (lead); resources (lead); supervision (lead); writing – original draft (lead). **Verónica Palma:** Conceptualization (lead); funding acquisition (lead); resources (lead); validation (lead); writing – original draft (lead).

## CONFLICT OF INTEREST

The authors have no conflicts of interest to declare.

### PEER REVIEW

The peer review history for this article is available at https://publons.com/publon/10.1002/btm2.10443.

## Supporting information


**Figure S1.** hWJCM is enriched in factors implicated in the regulation of angiogenesis.
**Figure S2.** Administration of hWJCM on Days 0, 5, and 7 concomitant to PD induction decreases the activation of γδ lymphocytes and, consequently, the levels of secreted IL‐17A in an IL‐17A reporter murine model.
**Figure S3.** Hyaluronic acid is able to reduce skin inflammation caused by topical application of IMQ in BALB/c mice.
**Figure S4.** Cartoon explaining criteria used for tortuous vessel quantification.
**Figure S5.** The splenic Index of mice induced with PD is significantly greater than that found in Ctrl mice, increasing in PD, hWJCM, HA Ctrl and HA hWJCM treatment groups.
**Table S1.** Body weight measurements of mice during all the long‐term PD induction conditions.
**Table S2.** Associations between Gene Ontology enrichment analysis involved in the immune response of hWJCM, as determined by an LC–MS/MS analysis.
**Table S3.** Summary of total proteins identified in the immunology pathway analysis in hWJCM.

## Data Availability

Author elects to not share data.
