# Supplementary material for: Efficacy of stem cell secretome loaded in hyaluronate sponge for topical treatment of psoriasis
Source: Bioeng Transl Med. 2023 Feb 21;8(2):e10443. doi: 10.1002/btm2.10443 (PMC10013801; doi:10.1002/btm2.10443)
Supplement: Supplementary file 1 — Figure S1. hWJCM is enriched in factors implicated in the regulation of angiogenesis. Figure S2. Administration of hWJCM on Days 0, 5, and 7 concomitant to PD induction decreases the activation of γδ lymphocytes and, consequently, the levels of secreted IL‐17A in an IL‐17A reporter murine model. Figure S3. Hyaluronic acid is able to reduce skin inflammation caused by topical application of IMQ in BALB/c mice. Figure S4. Cartoon explaining criteria used for tortuous vessel quantification. Figure S5. The splenic Index of mice induced with PD is significantly greater than that found in Ctrl mice, increasing in PD, hWJCM, HA Ctrl and HA hWJCM treatment groups. Table S1. Body weight measurements of mice during all the long‐term PD induction conditions. Table S2. Associations between Gene Ontology enrichment analysis involved in the immune response of hWJCM, as determined by an LC–MS/MS analysis. Table S3. Summary of total proteins identified in the immunology pathway analysis in hWJCM. [file BTM2-8-e10443-s001.docx]

**Supplementary materials***

**Figure S1.** hWJCM is enriched in factors implicated in the regulation of angiogenesis.

**Figure S2.** Administration of hWJCM on days 0, 5, and 7 concomitant to PD induction decreases the activation of γδ lymphocytes and, consequently, the levels of secreted IL-17A in an IL-17A reporter murine model.

**Figure S3.** Hyaluronic acid is able to reduce skin inflammation caused by topical application of IMQ in BALB/c mice.

**Figure S4.** Cartoon explaining criteria used for tortuous vessel quantification.

**Figure S5.** The splenic Index of mice induced with PD is significantly greater than that found in Ctrl mice, increasing in PD, hWJCM, HA Ctrl and HA hWJCM treatment groups.

**Table S1.** Body weight measurements of mice during all the long-term PD induction conditions.

**Table S2.** Associations between Gene Ontology enrichment analysis involved in the immune response of hWJCM, as determined by an LC-MS/MS analysis.

**Table S3.** Summary of total proteins identified in the immunology pathway analysis in hWJCM.

**
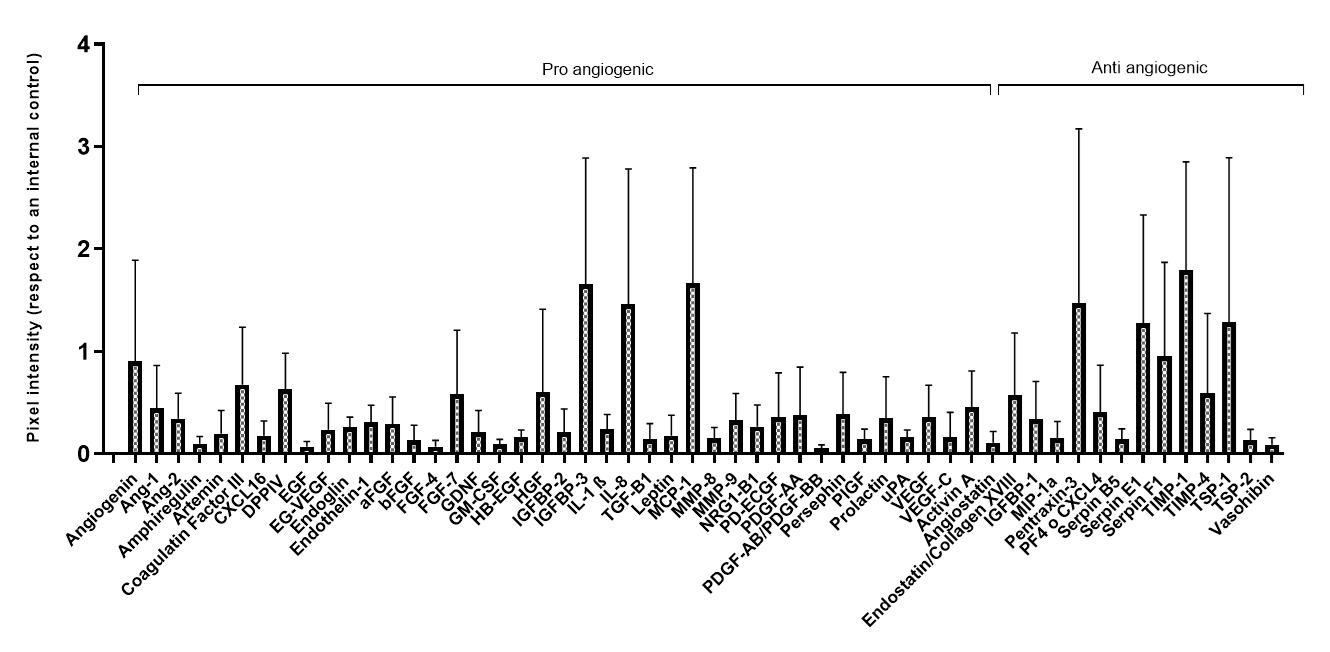
**

**Supplementary Figure 1. hWJCM obtained for this study is enriched in factors implicated in the regulation of angiogenesis.** The hWJ-MSC secretome contains both pro-angiogenic and anti-angiogenic molecules. The variability, expressed as standard deviation, is given by the inter-individual heterogeneity of the umbilical cords from which the hWJ-MSC and their conditioned media were isolated. Proteome profiler array, two-way ANOVA, multiple comparisons, Tukey test, α = 0.05, n=5.


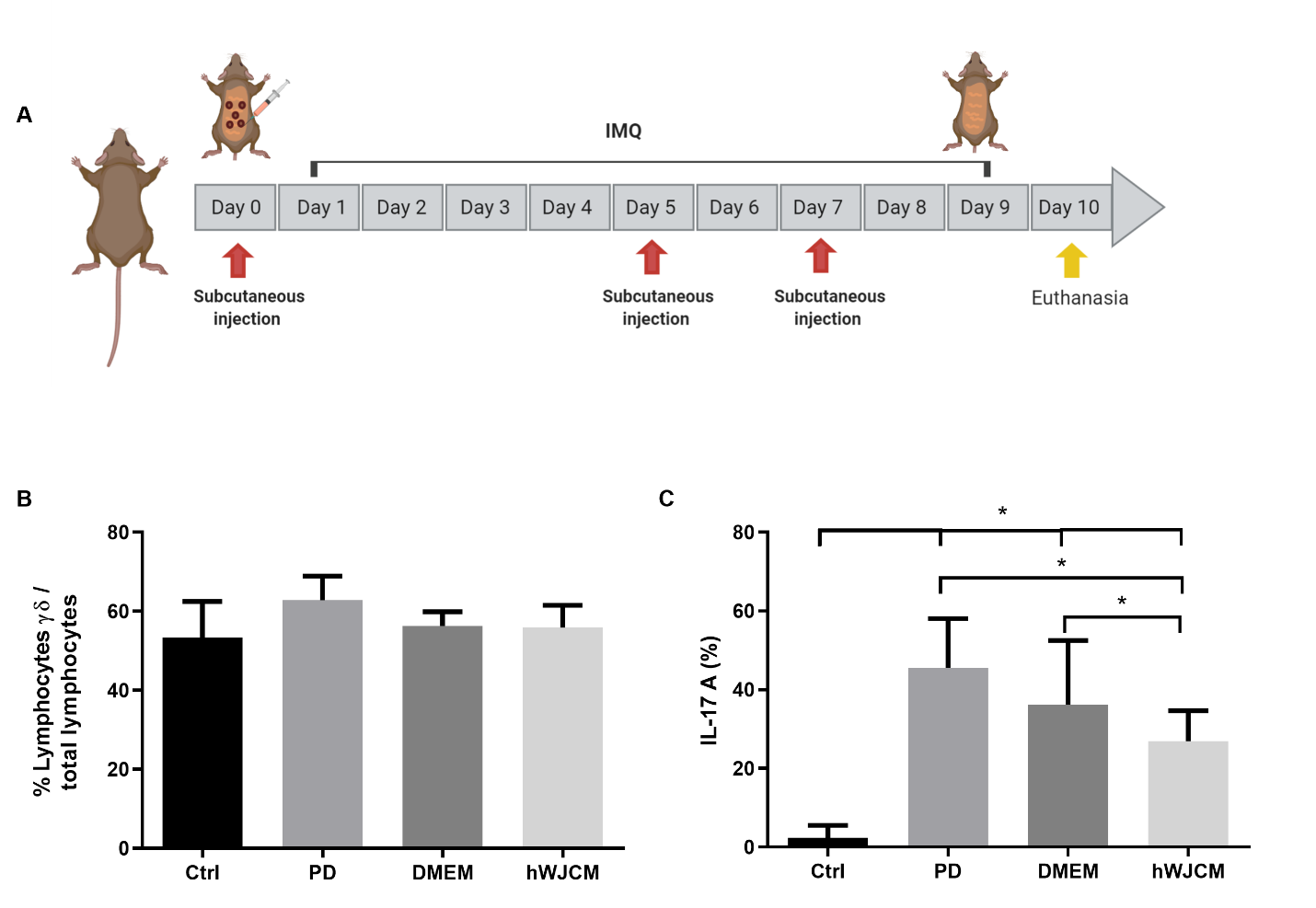


**Supplementary Figure 2. Administration of hWJCM on days 0, 5, and 7, concomitant to PD induction, decreases the activation of γδ lymphocytes and levels of secreted IL-17A** **in an IL-17A reporter murine model. (A)** Illustration of the experimental protocol. Created with BioRender.com **(B)** Comparison of the percentage of γδ T lymphocytes in freshly dissected skin samples at the end of the indicated treatments. **(C) Frequency of IL-17-producing cells within γδ T cells in harvested skin samples at day 10.** Data are presented as the mean ± SD and were analyzed using a paired permutation test, α = 0.05, n=3.


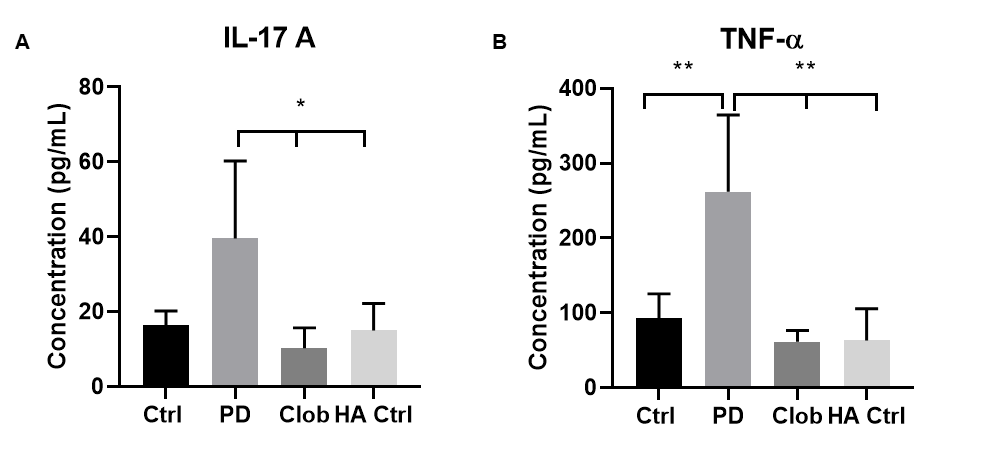


**Supplementary Figure 3. Hyaluronic acid is able to reduce skin inflammation caused by the topical application of IMQ in BALB/c mice.** Cytokines were measured after a 12-day PD induction treatment. **(A)** Concentration of IL-17A in Ctrl, PD, Clob and HA Ctrl groups. **(B)** Concentration of TNF-α in Ctrl, PD, Clob and HA Ctrl groups. One-way ANOVA, α = 0.05, n=4.


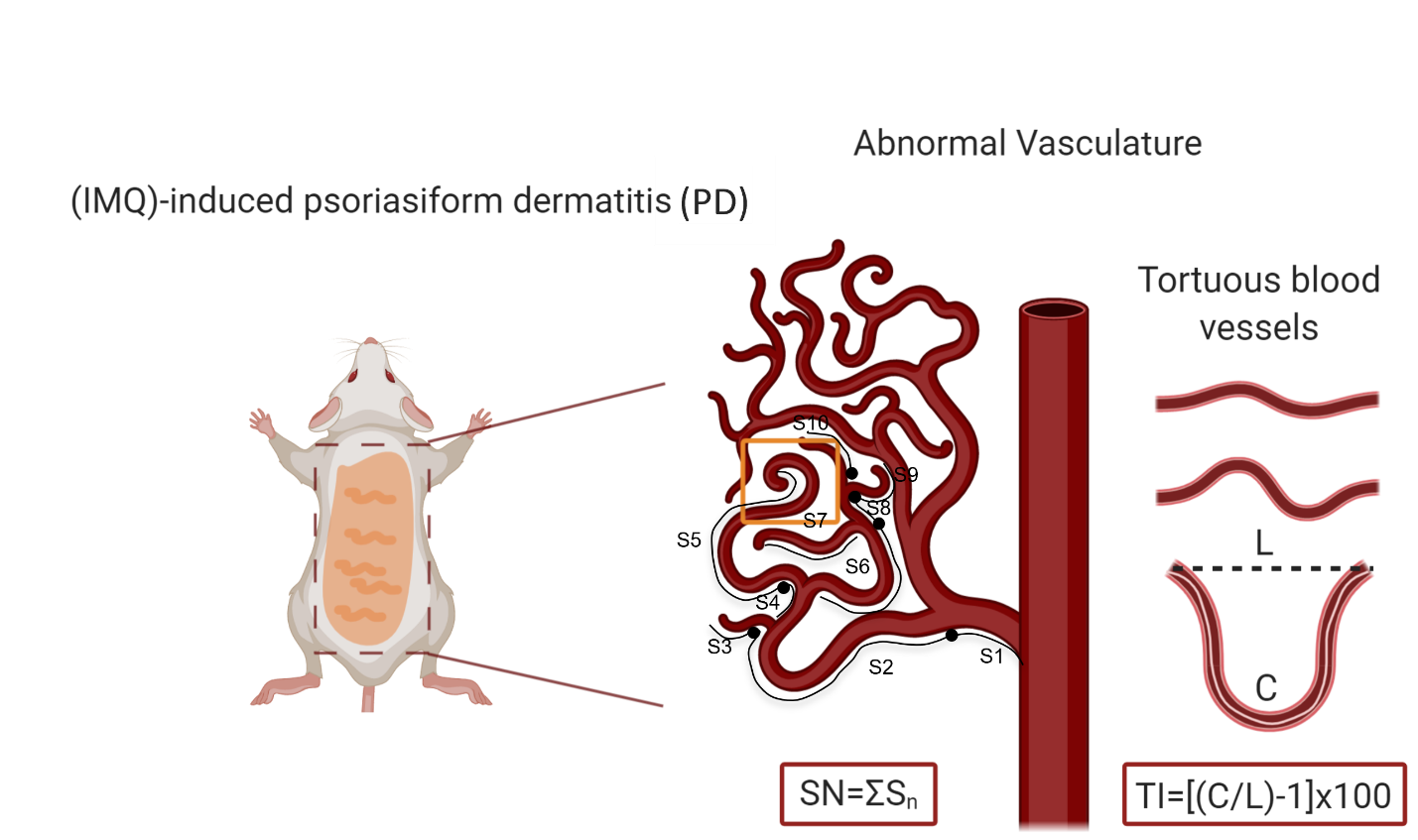


**Supplementary Figure 4. Cartoon explaining the criteria used for tortuous vessel quantification.** The dorsal skin of mice (4cm x 4 cm) was excised from each individual after treatment. Abnormal vasculature was considered as twisted or bent blood vessel architecture. In order to assess the tortuous complexity, the number of total segments (S1-S10) for the vessel was recorded based on Uylings & van Pelt criteria for a topological tree. As an example, a twisted blood vessel is highlighted by a square. Tortuosity index (TI) was used for the quantification of tortuosity, calculated as the ratio of curve length (C) over the shortest line connecting the starting and end point of a specific vessel (L), indicating the “level of coiliness” of the vessel. Created with BioRender.com.

**Supplementary Figure 5. The splenic Index of mice induced with PD is significantly greater than that found in the Ctrl mice.** Clob reduced the index, while hWJCM, HA Ctrl and HA hWJCM treatments displayed similar values to the PD condition. One-way ANOVA, α = 0.05, n = 3 (n=4 for Clob).

**
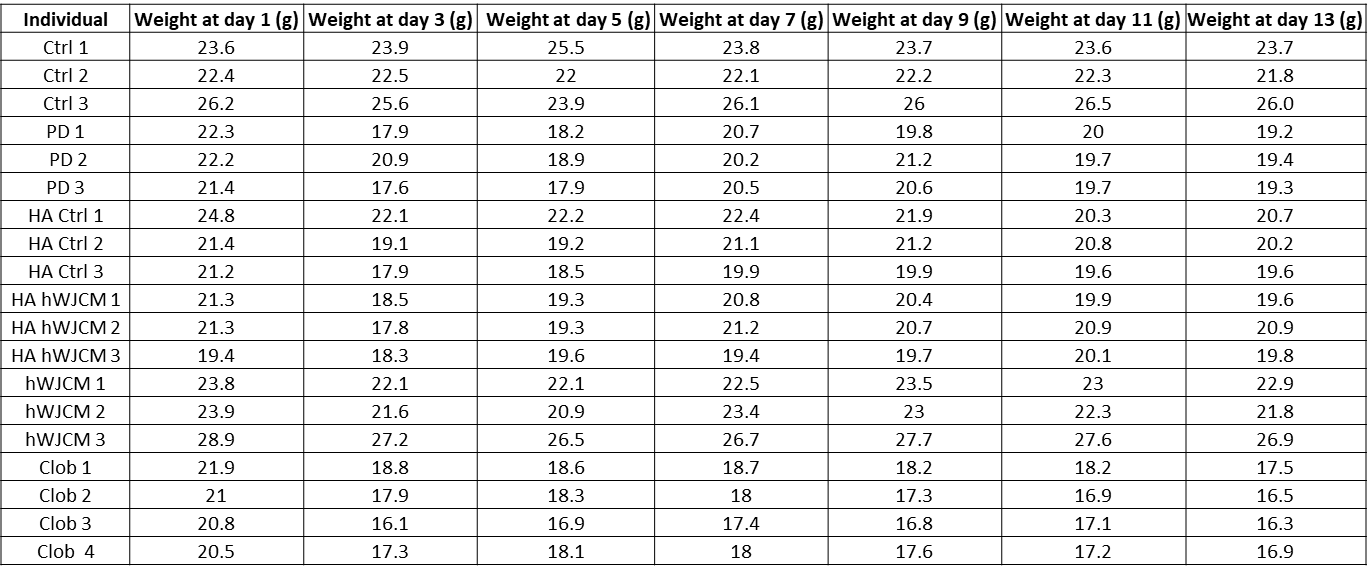
**

**Supplementary Table 1. Body weight measurement of BALB/c mice during long-term PD induction conditions.** Body weight was measured every other day during induction of PD, n=3, n=4 for Clob.

| **n°** | **Term ID** | **Term description** |
| --- | --- | --- |
| 1 | GO:0002376 | Immune system process (109) |
| 2 | GO:0002252 | Immune effector process (65) |
| 3 | GO:0002263 | Cell activation involved in immune response (51) |
| 4 | GO:0006955 | Immune response (78) |
| 5 | GO:0034097 | Response to cytokine (56) |
| 6 | GO:0002682 | Regulation of immune system process (64) |
| 7 | GO:0071345 | Cellular response to cytokine stimulus (50) |
| 8 | GO:0006954 | Inflammatory response (32) |
| 9 | GO:0050920 | Regulation of chemotaxis (18) |
| 10 | GO:0002684 | Positive regulation of immune system process (39) |
| 11 | GO:0050776 | Regulation of immune response (37) |
| 12 | GO:0002697 | Regulation of immune effector process (22) |
| 13 | GO:0002683 | Negative regulation of immune system process (22) |
| 14 | GO:0001819 | Positive regulation of cytokine production (21) |
| 15 | GO:0050727 | Regulation of inflammatory response (15) |
| 16 | GO:0046649 | Lymphocyte activation (16) |

**Supplementary Table 2. Associations between Gene Ontology enrichment analysis involved in the immune response of hWJCM, as determined by an LC-MS/MS analysis.** The total number of proteins found in each of these term descriptions related to immunological response is shown in parentheses. (n=4).

| **N°** | **Gene Names** | **% mín** | **% max** |  | **N°** | **Gene Names** | **% min** | **% max** |  | **N°** | **Gene Names** | **% min** | **% max** |
| --- | --- | --- | --- | --- | --- | --- | --- | --- | --- | --- | --- | --- | --- |
| **1** | A2M | 0,005% | 0,05% |  | **26** | CCL2 | 0,104% | 0,58% |  | **51** | EMILIN1 | 0,044% | 0,12% |
| **2** | ACTB | 0,545% | 1,11% |  | **27** | CD248 | 0,022% | 0,05% |  | **52** | FBN1 | 0,106% | 0,37% |
| **3** | ACTG1 | 0,545% | 1,11% |  | **28** | CD44 | 0,026% | 0,05% |  | **53** | FKBP1A | 0,139% | 0,66% |
| **4** | ACTN1 | 0,136% | 0,35% |  | **29** | CD59 | 0,144% | 0,30% |  | **54** | FLNA | 0,167% | 0,22% |
| **5** | ACTN4 | 0,098% | 0,32% |  | **30** | CDH13 | 0,014% | 0,04% |  | **55** | FLNB | 0,008% | 0,14% |
| **6** | ACTR2 | 0,023% | 0,11% |  | **31** | CFH | 0,122% | 0,22% |  | **56** | FLT1 | 0,033% | 0,06% |
| **7** | ACTR3 | 0,019% | 0,15% |  | **32** | CFL1 | 0,083% | 0,59% |  | **57** | FN1 | 0,284% | 0,58% |
| **8** | ADAM10 | 0,010% | 0,04% |  | **33** | CHI3L1 | 0,079% | 0,78% |  | **58** | FSCN1 | 0,048% | 0,12% |
| **9** | ADAM9 | 0,009% | 0,02% |  | **34** | CLU | 0,098% | 0,21% |  | **59** | FSTL3 | 0,107% | 0,44% |
| **10** | AHSG | 0,021% | 0,08% |  | **35** | COL1A1 | 0,370% | 1,60% |  | **60** | GALNT2 | 0,025% | 0,04% |
| **11** | ALDOA | 0,592% | 1,20% |  | **36** | COL1A2 | 0,529% | 1,71% |  | **61** | GAPDH | 0,095% | 0,27% |
| **12** | ANXA1 | 0,021% | 0,27% |  | **37** | COL2A1 | 0,007% | 0,04% |  | **62** | GAS6 | 0,039% | 0,10% |
| **13** | ANXA2 | 0,205% | 0,66% |  | **38** | COL3A1 | 0,169% | 0,73% |  | **63** | GGH | 0,009% | 0,02% |
| **14** | APP | 0,030% | 0,08% |  | **39** | COTL1 | 0,104% | 0,58% |  | **64** | GIG25 | 0,004% | 0,02% |
| **15** | ARPC1B | 0,022% | 0,14% |  | **40** | CST3 | 0,138% | 0,38% |  | **65** | GNS | 0,018% | 0,03% |
| **16** | ATRN | 0,003% | 0,01% |  | **41** | CTSB | 0,089% | 0,27% |  | **66** | GPC1 | 0,027% | 0,06% |
| **17** | AXL | 0,004% | 0,03% |  | **42** | CTSD | 0,038% | 0,07% |  | **67** | GPI | 0,045% | 0,09% |
| **18** | B2M | 0,111% | 0,38% |  | **43** | CTSZ | 0,076% | 0,13% |  | **68** | GREM1 | 0,052% | 0,54% |
| **19** | B4GALT1 | 0,015% | 0,05% |  | **44** | CXCL1 | 0,309% | 0,66% |  | **69** | GRN | 0,062% | 0,09% |
| **20** | BSG | 0,033% | 0,05% |  | **45** | CXCL8 | 0,309% | 0,58% |  | **70** | GSN | 0,044% | 0,08% |
| **21** | BST1 | 0,026% | 0,05% |  | **46** | DCD | 0,030% | 0,14% |  | **71** | HLA-B | 0,015% | 0,07% |
| **22** | C1R | 0,094% | 0,25% |  | **47** | DNASE2 | 0,016% | 0,08% |  | **72** | HRNR | 0,003% | 0,01% |
| **23** | C1S | 0,132% | 0,30% |  | **48** | DPP4 | 0,029% | 0,11% |  | **73** | HSPA5 | 0,087% | 0,33% |
| **24** | C3 | 0,075% | 0,28% |  | **49** | DSC1 | 0,002% | 0,01% |  | **74** | HSPA8 | 0,076% | 0,21% |
| **25** | CALR | 0,016% | 0,12% |  | **50** | ECM1 | 0,049% | 0,13% |  | **75** | HSPG2 | 0,079% | 0,13% |

| **N°** | **Gene Names** | **% min** | **% max** |  | **N°** | **Gene Names** | **% min** | **% max** |  | **N°** | **Gene Names** | **% min** | **% max** |
| --- | --- | --- | --- | --- | --- | --- | --- | --- | --- | --- | --- | --- | --- |
| **76** | HTRA1 | 0,050% | 0,18% |  | **101** | NME2 | 0,085% | 0,16% |  | **126** | SERPINE1 | 0,174% | 0,36% |
| **77** | ICAM1 | 0,014% | 0,03% |  | **102** | NPC2 | 0,269% | 0,40% |  | **127** | SERPINF1 | 0,021% | 0,10% |
| **78** | IDH1 | 0,008% | 0,07% |  | **103** | NPPB | 0,023% | 0,32% |  | **128** | SERPING1 | 0,008% | 0,05% |
| **79** | IGFBP4 | 0,182% | 0,58% |  | **104** | NRP1 | 0,005% | 0,02% |  | **129** | SOD1 | 0,085% | 0,22% |
| **80** | IL6 | 0,022% | 0,11% |  | **105** | NRP2 | 0,016% | 0,02% |  | **130** | SPARC | 0,263% | 1,20% |
| **81** | INHBA | 0,225% | 0,49% |  | **106** | P4HB | 0,104% | 0,29% |  | **131** | SPON2 | 0,104% | 0,22% |
| **82** | ITGB1 | 0,010% | 0,08% |  | **107** | PARK7 | 0,034% | 0,40% |  | **132** | SULF1 | 0,025% | 0,06% |
| **83** | KRT1 | 0,460% | 1,14% |  | **108** | PCOLCE | 0,034% | 0,21% |  | **133** | SULF2 | 0,002% | 0,01% |
| **84** | KRT16 | 0,106% | 0,38% |  | **109** | PDIA3 | 0,257% | 0,37% |  | **134** | TF | 0,002% | 0,04% |
| **85** | KRT6A | 0,165% | 0,99% |  | **110** | PKM | 0,097% | 0,31% |  | **135** | THBS1 | 0,165% | 0,33% |
| **86** | KRT8 | 0,251% | 0,92% |  | **111** | PLAUR | 0,025% | 0,04% |  | **136** | TIMP1 | 0,309% | 0,56% |
| **87** | LAMP1 | 0,012% | 0,02% |  | **112** | POSTN | 0,205% | 0,35% |  | **137** | TIMP2 | 0,382% | 0,66% |
| **88** | LGALS1 | 0,409% | 0,65% |  | **113** | PPIA | 0,166% | 0,35% |  | **138** | TMSB4X | 6,074% | 31,47% |
| **89** | LOX | 0,209% | 0,54% |  | **114** | PRDX1 | 0,299% | 0,89% |  | **139** | TNFRSF12A | 0,058% | 0,10% |
| **90** | LRP1 | 0,003% | 0,02% |  | **115** | PRDX2 | 0,063% | 0,16% |  | **140** | TXNDC17 | 0,040% | 0,07% |
| **91** | LTF | 0,007% | 0,02% |  | **116** | PRNP | 0,009% | 0,09% |  | **141** | TXNDC5 | 0,100% | 0,16% |
| **92** | LUM | 0,165% | 0,42% |  | **117** | PSAP | 0,102% | 0,17% |  | **142** | VCL | 0,026% | 0,09% |
| **93** | MANBA | 0,018% | 0,05% |  | **118** | PTX3 | 0,102% | 0,18% |  | **143** | VIM | 0,466% | 2,09% |
| **94** | MDK | 0,118% | 0,17% |  | **119** | PVR | 0,027% | 0,06% |  | **144** | VTN | 0,004% | 0,06% |
| **95** | MMP1 | 0,007% | 0,23% |  | **120** | PXDN | 0,112% | 0,24% |  | **145** | WDR1 | 0,022% | 0,10% |
| **96** | MMP2 | 0,329% | 0,74% |  | **121** | QSOX1 | 0,074% | 0,12% |  | **146** | YBX1 | 0,032% | 0,09% |
| **97** | MSN | 0,117% | 0,69% |  | **122** | RTN4 | 0,004% | 0,01% |  | **147** | YWHAZ | 0,049% | 0,40% |
| **98** | MT2A | 0,706% | 3,66% |  | **123** | S100A11 | 0,242% | 0,49% |  | **148** | ZYX | 0,036% | 0,15% |
| **99** | MUCL1 | 0,040% | 0,21% |  | **124** | SCG2 | 0,020% | 0,18% |  |  |  |  |  |
| **100** | MYH9 | 0,006% | 0,60% |  | **125** | SEMA7A | 0,033% | 0,05% |  |  |  |  |  |

**Supplementary Table 3. Summary of total proteins identified in the immunology pathway analysis in hWJCM.** The relative abundance of the 148 proteins involved in immune pathways, as determined by LC-MS/MS analysis and referred to Suppl. table 2, is listed. The list is alphabetically ordered by gene names referring in each case to protein content indicating the minimum and maximum percentage belonging to each of the four hWJCM samples.
